# Diazido­bis(propane-1,3-diamine)copper(II)

**DOI:** 10.1107/S1600536810010184

**Published:** 2010-03-24

**Authors:** Islam Ullah Khan, Ejaz, Onur Şahin, Orhan Büyükgüngör

**Affiliations:** a Materials Chemistry Laboratry, Department of Chemistry, GC University, Lahore 54000, Pakistan; b Department of Physics, Ondokuz Mayıs University, TR-55139 Samsun, Turkey

## Abstract

In the title complex, [Cu(N_3_)_2_(C_3_H_10_N_2_)_2_], the Cu^II^ ion resides on a centre of symmetry and is in a Jahn–Teller distorted octa­hedral coordination environment comprising two N atoms from azide anions in axial positions and four N atoms from propane-1,3-diamine (tn) ligands in equatorial positions. Inter­molecular N—H⋯N hydrogen bonds produce *R*_2_^1^(6), *R*_2_^2^(8), *R*_2_^2^(12) and *R*_4_^2^(8) rings, generating a two-dimensional layer.

## Related literature

For related structures, see: Escuer *et al.* (1997 ▶); Gu *et al.* (2007 ▶); Mondal & Mukherjee (2008 ▶); Monfort *et al.* (2000 ▶); Shen *et al.* (2000 ▶); Sundberg & Sillanpaa (1993 ▶); Sundberg & Uggla (1997 ▶); Sundberg *et al.* (2001 ▶); Zhang *et al.* (2009 ▶); Luo *et al.* (2004 ▶); Triki *et al.* (2005 ▶). For graph-set motifs, see: Bernstein *et al.* (1995 ▶).
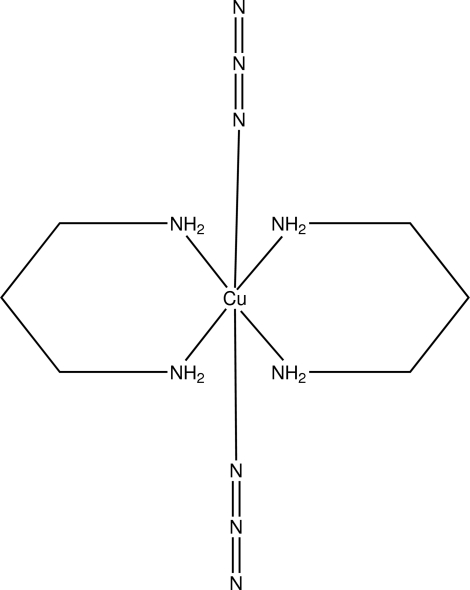


## Experimental

### 

#### Crystal data

[Cu(N_3_)_2_(C_3_H_10_N_2_)_2_]
*M* *_r_* = 295.86
Triclinic, 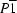

*a* = 6.6869 (4) Å
*b* = 6.7743 (4) Å
*c* = 8.2445 (8) Å
α = 93.296 (3)°
β = 98.306 (3)°
γ = 119.453 (2)°
*V* = 318.19 (4) Å^3^
*Z* = 1
Mo *K*α radiation
μ = 1.72 mm^−1^
*T* = 296 K
0.27 × 0.25 × 0.22 mm

#### Data collection

Bruker Kappa APEXII diffractometer
5360 measured reflections
1497 independent reflections
1467 reflections with *I* > 2σ(*I*)
*R*_int_ = 0.023

#### Refinement

*R*[*F* ^2^ > 2σ(*F* ^2^)] = 0.018
*wR*(*F* ^2^) = 0.077
*S* = 1.01
1497 reflections
95 parameters
4 restraints
H atoms treated by a mixture of independent and constrained refinement
Δρ_max_ = 0.42 e Å^−3^
Δρ_min_ = −0.44 e Å^−3^

### 

Data collection: *APEX2* (Bruker, 2009 ▶); cell refinement: *SAINT* (Bruker, 2009 ▶); data reduction: *SAINT*; program(s) used to solve structure: *SHELXS97* (Sheldrick, 2008 ▶); program(s) used to refine structure: *SHELXL97* (Sheldrick, 2008 ▶); molecular graphics: *ORTEP-3 for Windows* (Farrugia, 1997 ▶); software used to prepare material for publication: *WinGX* (Farrugia, 1999 ▶).

## Supplementary Material

Crystal structure: contains datablocks global, I. DOI: 10.1107/S1600536810010184/om2325sup1.cif

Structure factors: contains datablocks I. DOI: 10.1107/S1600536810010184/om2325Isup2.hkl

Additional supplementary materials:  crystallographic information; 3D view; checkCIF report

## Figures and Tables

**Table:** 

N1—Cu1	2.0333 (13)
N2—Cu1	2.0302 (13)
N3—N5	1.169 (2)
N4—N5	1.168 (2)
N4—Cu1	2.6740 (17)

**Table:** 

N5—N4—Cu1	99.05 (12)
N4—N5—N3	179.8 (2)
N2—Cu1—N1	87.19 (5)
N2—Cu1—N4	83.92 (5)
N1—Cu1—N4	87.19 (5)

**Table 2:** Hydrogen-bond geometry (Å, °)

*D*—H⋯*A*	*D*—H	H⋯*A*	*D*⋯*A*	*D*—H⋯*A*
N1—H1⋯N3^i^	0.84 (1)	2.12 (2)	2.962 (2)	173 (2)
N1—H2⋯N4^ii^	0.85 (2)	2.66 (2)	3.511 (2)	173 (2)
N2—H3⋯N3^ii^	0.83 (2)	2.44 (2)	3.220 (2)	158 (2)
N2—H4⋯N3^iii^	0.80 (2)	2.31 (2)	3.078 (2)	162 (2)

Symmetry codes: (i) 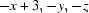; (ii) 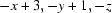; (iii) 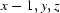.

**Fig. 1.**
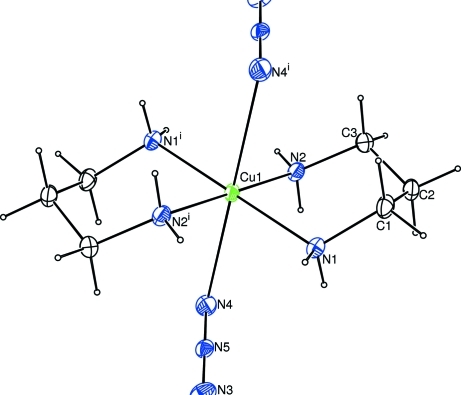
A view of one molecule showing the atom-numbering scheme. Displacement ellipsoids are drawn at the 30% probability level and H atoms are shown as small spheres of arbitrary radii. [Symmetry code: (i) 2-x, -y, -z.]

**Fig. 2.**
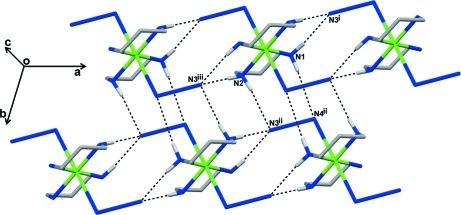
Part of the crystal structure showing the formation of R21(6), R22(8), R22(12) and R42(8) rings. H atoms not involved in these interactions have been omitted for clarity. (Symmetry codes as in Table 2).

## supplementary crystallographic information

### Comment

Recently, metal azide complexes have attracted great attention (Mondal & Mukherjee, 2008; Gu *et al.*, 2007). The azide anion has rich coordination modes (Shen *et al.*, 2000), and many metal-azide complexes have been reported (Monfort *et al.*, 2000). In most of the compounds reported to date, the co-ligands are neutral organic ligands, while charged ligands are very scarce (Escuer *et al.*, 1997). The 1,3-diaminopropane (tn) ligand behaves as a strong chelatator in its metal complexes due to the formation of a stable six-membered ring. At the same time, it is a good H-bond donor due to the existence of amino groups (Sundberg *et al.*, 2001). Previously, the polymorphic dinuclear compound featuring both bridging and terminal azido groups was reported (Luo *et al.*, 2004; Triki *et al.*,  2005). Herein, we report the synthesis and structure of the mononuclear complex with only terminal azido ligands.

The molecular structure and atom-labelling scheme are shown in Fig. 1. The Cu^II^ atom is located on a center of symmetry and is coordinated by four N atoms from two tn ligands and two N atoms from two azide anions. The geometry around the Cu^II^ ion (Table 1) is that of a distorted octahedron, the equatorial plane of which (N1/N2/N1^i^/N2^i^) is formed by four amino N atoms [symmetry code: (i) 2-x, -y, -z]. The axial positions in the octahedron are occupied by two N atoms (N4 and N4^i^). The Cu1—N4 distance is longer than the corresponding distances in related structures (Luo *et al.*, 2004; Triki *et al.*, 2005). This elongation can be attributed to the static Jahn-Teller effect. The tn ligand shows chelating coordination behavior and displays a chair conformation in the equatorial direction. This kind of coordination mode was also found in the similar complexes (Sundberg *et al.*, 2001; Sundberg & Sillanpaa, 1993; Sundberg & Uggla, 1997). The Cu1—N1 and Cu1—N2 bond lengths are very similar to those in the previously reported Bis(4-aminobenzenesulfonato-κ*O*)bis(propane-1,3-diamine-κ^2^*N*,\ N')copper(II) dihydrate (Zhang *et al.*, 2009).

Amino atom N2 in the molecule at (x, y, z) acts as a hydrogen-bond donor, via H3, to atom N3^ii^ so forming a C(6) (Bernstein *et al.*, 1995) chain running parallel to the [110] direction. Amino atom N2 in the molecule at (x, y, z) acts as a hydrogen-bond donor, via H4, to atom N3^iii^ so forming a C(6) chain running parallel to the [-100] direction. Similarly, amino atom N1 in the molecule at (x, y, z) acts as a hydrogen-bond donor, via H1, to atom N3^i^ so forming a C(6) chain running parallel to the [100] direction. Amino atom N1 in the molecule at (x, y, z) acts as a hydrogen-bond donor, via H2, to atom N4^ii^ so forming a C(4) chain running parallel to the [110] direction. The combination of C(4) and C(6) chains produce R_2_^1^(6), R_2_^2^(8), R_2_^2^(12) and R_4_^2^(8) rings (Fig. 2).

### Experimental

Copper(II) sulphate (0.16 g, 1.0 mmol) was dissolved in methanol (20 ml). Sodium azide (0.134 g, 2.0 mmol) and 1,3-diaminopropane(0.148 g, 2.0 mmol) were added and the mixture refluxed for 3 hours. A blue solution formed, which was filtered. After a few days, blue blocks were obtained from the methanol filtrate.

### Refinement

All H atoms bound to C atoms were refined using a riding model, with C—H = 0.97Å and U_iso_(H) = 1.2U_eq_(C) for methylene C atoms. Amino H atoms were located in difference maps and refined subject to a DFIX restraint of N—H = 0.87 (2) Å.

### Crystal data

**Table:**

[Cu(N_3_)_2_(C_3_H_10_N_2_)_2_]	*Z* = 1
*M**_r_* = 295.86	*F*(000) = 155
Triclinic, *P*1	*D*_x_ = 1.544 Mg m^−^^3^
Hall symbol: -P 1	Mo *K*α radiation, λ = 0.71073 Å
*a* = 6.6869 (4) Å	Cell parameters from 4650 reflections
*b* = 6.7743 (4) Å	θ = 3.5–28.6°
*c* = 8.2445 (8) Å	µ = 1.72 mm^−^^1^
α = 93.296 (3)°	*T* = 296 K
β = 98.306 (3)°	Blocks, blue
γ = 119.453 (2)°	0.27 × 0.25 × 0.22 mm
*V* = 318.19 (4) Å^3^	

### Data collection

**Table:**

Bruker Kappa APEXII diffractometer	1467 reflections with *I* > 2σ(*I*)
Radiation source: fine-focus sealed tube	*R*_int_ = 0.023
graphite	θ_max_ = 28.0°, θ_min_ = 2.5°
φ and ω scans	*h* = −8→5
5360 measured reflections	*k* = −8→8
1497 independent reflections	*l* = −10→10

### Refinement

**Table:**

Refinement on *F*^2^	Primary atom site location: structure-invariant direct methods
Least-squares matrix: full	Secondary atom site location: difference Fourier map
*R*[*F*^2^ > 2σ(*F*^2^)] = 0.018	Hydrogen site location: inferred from neighbouring sites
*wR*(*F*^2^) = 0.077	H atoms treated by a mixture of independent and constrained refinement
*S* = 1.01	*w* = 1/[σ^2^(*F*_o_^2^) + (0.0676*P*)^2^ + 0.0082*P*] where *P* = (*F*_o_^2^ + 2*F*_c_^2^)/3
1497 reflections	(Δ/σ)_max_ < 0.001
95 parameters	Δρ_max_ = 0.42 e Å^−^^3^
4 restraints	Δρ_min_ = −0.44 e Å^−^^3^

### Special details

**Table:**

Geometry. All esds (except the esd in the dihedral angle between two l.s. planes) are estimated using the full covariance matrix. The cell esds are taken into account individually in the estimation of esds in distances, angles and torsion angles; correlations between esds in cell parameters are only used when they are defined by crystal symmetry. An approximate (isotropic) treatment of cell esds is used for estimating esds involving l.s. planes.
Refinement. Refinement of *F*^2^ against ALL reflections. The weighted *R*-factor wR and goodness of fit *S* are based on *F*^2^, conventional *R*-factors *R* are based on *F*, with *F* set to zero for negative *F*^2^. The threshold expression of *F*^2^ > σ(*F*^2^) is used only for calculating *R*-factors(gt) etc. and is not relevant to the choice of reflections for refinement. *R*-factors based on *F*^2^ are statistically about twice as large as those based on *F*, and *R*- factors based on ALL data will be even larger.

### Fractional atomic coordinates and isotropic or equivalent isotropic displacement parameters (Å^2^)

**Table:**

	*x*	*y*	*z*	*U*_iso_*/*U*_eq_	
C1	1.2523 (3)	0.1281 (3)	0.3530 (2)	0.0388 (4)	
H1A	1.3929	0.1612	0.4300	0.047*	
H1B	1.1251	−0.0175	0.3700	0.047*	
C2	1.1953 (3)	0.3138 (3)	0.3892 (2)	0.0401 (4)	
H2A	1.3207	0.4574	0.3679	0.048*	
H2B	1.1916	0.3312	0.5062	0.048*	
C3	0.9655 (3)	0.2714 (3)	0.2899 (2)	0.0400 (4)	
H3A	0.8381	0.1311	0.3136	0.048*	
H3B	0.9412	0.3964	0.3233	0.048*	
N1	1.2889 (2)	0.1090 (2)	0.18062 (17)	0.0313 (3)	
H1	1.338 (3)	0.017 (3)	0.173 (2)	0.029 (5)*	
H2	1.398 (3)	0.241 (3)	0.169 (3)	0.034 (5)*	
N2	0.9619 (2)	0.2524 (2)	0.10959 (17)	0.0317 (3)	
H3	1.078 (3)	0.368 (3)	0.093 (3)	0.037 (5)*	
H4	0.855 (3)	0.261 (4)	0.060 (3)	0.040 (6)*	
N3	1.5787 (3)	0.2474 (3)	−0.1503 (2)	0.0483 (4)	
N4	1.2752 (3)	0.3278 (3)	−0.1626 (2)	0.0446 (3)	
N5	1.4271 (2)	0.2879 (2)	−0.15632 (16)	0.0310 (3)	
Cu1	1.0000	0.0000	0.0000	0.02769 (12)	

### Atomic displacement parameters (Å^2^)

**Table:**

	*U*^11^	*U*^22^	*U*^33^	*U*^12^	*U*^13^	*U*^23^
C1	0.0421 (9)	0.0354 (8)	0.0248 (8)	0.0114 (7)	−0.0028 (6)	0.0058 (6)
C2	0.0409 (9)	0.0375 (8)	0.0246 (7)	0.0085 (7)	0.0031 (6)	−0.0030 (6)
C3	0.0374 (8)	0.0407 (9)	0.0331 (8)	0.0141 (7)	0.0078 (7)	−0.0065 (7)
N1	0.0284 (6)	0.0309 (6)	0.0293 (6)	0.0132 (5)	−0.0013 (5)	0.0012 (5)
N2	0.0278 (6)	0.0320 (7)	0.0304 (7)	0.0136 (5)	−0.0001 (5)	−0.0004 (5)
N3	0.0379 (8)	0.0398 (8)	0.0659 (11)	0.0211 (7)	0.0029 (7)	0.0040 (7)
N4	0.0365 (7)	0.0575 (9)	0.0397 (8)	0.0255 (7)	0.0029 (6)	0.0027 (7)
N5	0.0288 (6)	0.0254 (6)	0.0294 (6)	0.0069 (5)	0.0043 (5)	0.0056 (5)
Cu1	0.02519 (16)	0.03367 (17)	0.02129 (17)	0.01466 (12)	−0.00046 (10)	−0.00212 (10)

### Geometric parameters (Å, °)

**Table:**

C1—N1	1.486 (2)	N1—H1	0.843 (14)
C1—C2	1.509 (3)	N1—H2	0.852 (15)
C1—H1A	0.9700	N2—Cu1	2.0302 (13)
C1—H1B	0.9700	N2—H3	0.826 (16)
C2—C3	1.513 (2)	N2—H4	0.801 (15)
C2—H2A	0.9700	N3—N5	1.169 (2)
C2—H2B	0.9700	N4—N5	1.168 (2)
C3—N2	1.480 (2)	N4—Cu1	2.6740 (17)
C3—H3A	0.9700	Cu1—N2^i^	2.0302 (13)
C3—H3B	0.9700	Cu1—N1^i^	2.0333 (13)
N1—Cu1	2.0333 (13)		
			
N1—C1—C2	111.99 (13)	C1—N1—H2	106.5 (15)
N1—C1—H1A	109.2	Cu1—N1—H2	110.7 (14)
C2—C1—H1A	109.2	H1—N1—H2	108.1 (19)
N1—C1—H1B	109.2	C3—N2—Cu1	118.90 (11)
C2—C1—H1B	109.2	C3—N2—H3	108.0 (15)
H1A—C1—H1B	107.9	Cu1—N2—H3	101.3 (15)
C1—C2—C3	114.90 (15)	C3—N2—H4	110.7 (17)
C1—C2—H2A	108.5	Cu1—N2—H4	113.0 (17)
C3—C2—H2A	108.5	H3—N2—H4	103 (2)
C1—C2—H2B	108.5	N5—N4—Cu1	99.05 (12)
C3—C2—H2B	108.5	N4—N5—N3	179.8 (2)
H2A—C2—H2B	107.5	N2—Cu1—N2^i^	180.00 (7)
N2—C3—C2	111.68 (13)	N2—Cu1—N1	87.19 (5)
N2—C3—H3A	109.3	N2^i^—Cu1—N1	92.81 (5)
C2—C3—H3A	109.3	N2—Cu1—N1^i^	92.81 (5)
N2—C3—H3B	109.3	N2^i^—Cu1—N1^i^	87.19 (5)
C2—C3—H3B	109.3	N1—Cu1—N1^i^	180.00 (6)
H3A—C3—H3B	107.9	N2—Cu1—N4	83.92 (5)
C1—N1—Cu1	115.28 (10)	N2^i^—Cu1—N4	96.08 (5)
C1—N1—H1	107.0 (13)	N1—Cu1—N4	87.19 (5)
Cu1—N1—H1	109.1 (14)	N1^i^—Cu1—N4	92.81 (5)
			
N1—C1—C2—C3	64.96 (19)	C1—N1—Cu1—N2	52.35 (11)
C1—C2—C3—N2	−60.6 (2)	C1—N1—Cu1—N2^i^	−127.65 (11)
C2—C1—N1—Cu1	−66.39 (15)	C1—N1—Cu1—N4	136.40 (11)
C2—C3—N2—Cu1	60.49 (17)	N5—N4—Cu1—N2	137.75 (12)
C3—N2—Cu1—N1	−50.92 (12)	N5—N4—Cu1—N2^i^	−42.25 (12)
C3—N2—Cu1—N1^i^	129.08 (12)	N5—N4—Cu1—N1	50.28 (12)
C3—N2—Cu1—N4	−138.39 (12)	N5—N4—Cu1—N1^i^	−129.72 (12)

Symmetry codes: (i) −*x*+2, −*y*, −*z*.

### Hydrogen-bond geometry (Å, °)

**Table:**

*D*—H···*A*	*D*—H	H···*A*	*D*···*A*	*D*—H···*A*
N1—H1···N3^ii^	0.84 (1)	2.12 (2)	2.962 (2)	173 (2)
N1—H2···N4^iii^	0.85 (2)	2.66 (2)	3.511 (2)	173 (2)
N2—H3···N3^iii^	0.83 (2)	2.44 (2)	3.220 (2)	158 (2)
N2—H4···N3^iv^	0.80 (2)	2.31 (2)	3.078 (2)	162 (2)

Symmetry codes: (ii) −*x*+3, −*y*, −*z*; (iii) −*x*+3, −*y*+1, −*z*; (iv) *x*−1, *y*, *z*.
